# Legacy Pb–Zn mine tailings as persistent sources of bioaccessible potentially toxic elements in urban dust: geochemical signatures, exposure pathways, and human health risks

**DOI:** 10.1007/s10661-026-15743-x

**Published:** 2026-08-13

**Authors:** Edvaldo Renner da Costa Cardoso, Rugana Imbaná, Ésio de Castro Paes, Isabela C. F. Vasques, Oldair Vinhas Costa, Thomas Vincent Gloaguen, Emanuelle Mercês Barros Soares

**Affiliations:** 1 Department of Soils, Federal University of Viçosa, R. Purdue S/N, Campus Universitário, Viçosa, Minas Gerais 36570-900 Brazil; 2 Center for Agricultural, Environmental and Biological Sciences, Federal University of Recôncavo of Bahia, Cruz das Almas, 44380-000 Brazil

**Keywords:** Atmospheric pollution, Hazard index, Source apportionment, Bioaccessibility

## Abstract

Abandoned mine tailings, rich in potentially toxic elements (PTEs), can be redistributed in urban areas through atmospheric resuspension. This process serves as a dynamic geochemical compartment linking legacy contamination to human exposure. This study investigated the occurrence, oral bioaccessibility, ecological significance, and human health risks of PTEs in urban dust from Boquira, a former Pb–Zn mining district in northeastern Brazil. Eighteen urban dust samples and reference soils from uncontaminated areas were collected and analyzed for pseudo-total concentrations of Pb, Zn, Cd, Cu, Mn, Fe, and Al. Analyses were conducted using microwave-assisted acid digestion and X-ray fluorescence spectrometry (XRF). Geochemical indices, including the geoaccumulation index (I_geo_), contamination factor (CF), enrichment factor (EF), and ecological risk index (Er), were used to evaluate anthropogenic enrichment and its ecological implications. Oral bioaccessibility was assessed using a simplified dilute nitric acid extraction method that simulates gastric conditions. Elevated enrichment and contamination indices revealed substantial anthropogenic inputs, particularly for Pb and Zn, indicating the long-term environmental persistence of mining-derived particulates. Pb had the highest mean oral bioaccessibility (~ 75%), followed by Cu (54.8%), Mn (28.1%), and Zn (27.0%), indicating the predominance of highly reactive and physiologically available metal-bearing phases. Human health risk assessment identified oral ingestion as the dominant exposure pathway, compared to dermal exposure, with children presenting substantially higher risks than adults. Non-carcinogenic risk thresholds (HI > 1) were exceeded for children, primarily due to Pb exposure, whereas carcinogenic risk associated with Cd ingestion exceeded acceptable limits for both population groups. The results demonstrate that legacy mine tailings in Boquira remain environmentally active decades after mine closure, sustaining chronic atmospheric redistribution of bioaccessible PTEs into urban areas.

**Supplementary Information:**

The online version contains supplementary material available at https://doi.org/10.1007/s10661-026-15743-x.

## Introduction

Potentially toxic elements (PTEs) are pervasive environmental contaminants whose accumulation reflects the increasing intensity of human disturbance in terrestrial systems. While naturally present in the pedosphere, their enrichment in soils, sediments, and atmospheric particulates is significantly amplified by industrial activities, particularly mining (Cheng et al., 2020; Paes et al., 2022, a, b, 2023c; Zhong et al., 2020). In post-mining landscapes, these elements persist long after operational closure, forming legacy contaminants that continuously interact with surface processes and human environments. Urban dust is one of the easiest ways PTEs reach houses (Ediagbonya et al., 2026), especially in areas close to mine tailings (Zanetta-Colombo et al., 2022). The heterogeneity of urban environments and their division into functional zones, such as commercial and residential, makes the evaluation of the contamination scenario very challenging (Huang et al., 2026).

Evaluating the extent and origin of PTE contamination remains a pivotal challenge in environmental geochemistry. Widely employed geochemical indices, such as the geoaccumulation index (Igeo), enrichment factor (EF), contamination factor (CF), and potential ecological risk index (ER), provide valuable yet inherently limited metrics of contamination and ecological risk (Nahum et al., 2024; Setu & Strezov, 2025). Their interpretation is critically dependent on background values, which are often uncertain in mining-affected terrains where natural and anthropogenic signals overlap (Firmino et al., 2025). Emerging approaches based on statistically derived baselines from major elements offer enhanced resolution by accounting for geological variability and enabling more robust discrimination of contamination sources (Matschullat et al., 2000; Reimann & Garrett, 2005).

Human exposure to PTEs is mediated through intricate environmental pathways, with urban dust serving as a pivotal yet frequently overlooked interface. In mining regions, fine particles originating from tailings can be transported, deposited, and resuspended within urban settings, thereby facilitating exposure through ingestion, inhalation, and dermal contact (Bartholomew et al., 2020; Lima & Bernardez, 2017). Notably, the toxicity of these PTEs depends not only on their total concentration but also on their bioaccessible fraction, which represents the proportion that potentially becomes soluble under physiological conditions and is consequently available for absorption. In vitro bioaccessibility tests, employing synthetic fluids that emulate gastrointestinal environments, have emerged as indispensable tools for bridging the gap between environmental contamination and human health risk (Ettler et al., 2019; Intawongse & Dean, 2006; Ren et al., 2020).

Although bioavailability can be directly assessed through in vivo approaches, bioaccessibility provides a tractable and informative proxy for exposure assessment (Kastury et al., 2017; Ren et al., 2020). Established methods such as the physiologically based extraction test (PBET), In Vitro Gastrointestinal (IVG), and the Unified BARGE Method (UBM), among others, simulate gastrointestinal conditions with increasing complexity (Denys et al., 2012; Rodriguez et al., 1999; Ruby et al., 1996; Wragg & Klinck, 2007), yet their application is often limited by analytical costs and infrastructure constraints. Simplified extraction procedures using diluted inorganic solutions have demonstrated strong correlations with these standardized protocols and offer a pragmatic alternative for large-scale screening (Rodrigues et al., 2018).

Integrating bioaccessible concentrations with exposure models facilitates the quantitative estimation of human health risks. Metrics such as the hazard index (HI) and carcinogenic risk (CR) provide standardized frameworks for assessing the likelihood of adverse effects. Thresholds (HI > 1; CR > 1 × 10^–4^) indicate potential concern (Kan et al., 2021). These approaches have been extensively applied in post-mining and post-disaster contexts, consistently demonstrating elevated risks associated with PTE exposure in contaminated environments (Davila et al., 2020; Gomes et al., 2022; Paoliello et al., 2002; Santos et al., 2023).

In northeastern Brazil, the municipality of Boquira serves as a case study of a legacy mining landscape characterized by enduring environmental contamination even decades after mine closure. An abandoned tailings pile, enriched in lead (Pb) and zinc (Zn), remains exposed and continues to emit particulate matter. The region is situated within the Paramirim Valley, a geological domain naturally endowed with metallic elements, including Pb and Cd (Carvalho et al., 1982; Gomes et al., 2020). This natural enrichment complicates the distinction between natural and anthropogenic signals. While previous studies have primarily focused on soil and mining residues (Paes et al., 2022, 2023a, b, 2025), the role of urban and domestic dust as an exposure pathway remains inadequately understood. This is particularly evident when compared to more extensively studied systems, such as Santo Amaro (Amorim et al., 2021; Silva et al., 2017).

In this study, we quantify the total and bioaccessible concentrations of PTEs in urban dust from Boquira. We employ geochemical indices to assess contamination levels and evaluate the associated human health risks through ingestion and dermal exposure pathways. By employing a simplified nitric acid extraction method, we estimate the oral bioaccessibility of PTEs, focusing on the fraction most relevant to human exposure. Our hypothesis posits that Boquira’s mine tailings serve as a primary source of PTE-enriched dust in surrounding residential areas, constituting a persistent and underrecognized pathway of environmental contamination and human risk.

## Material and methods

### Study area and sample collection

**Fig. 1:**
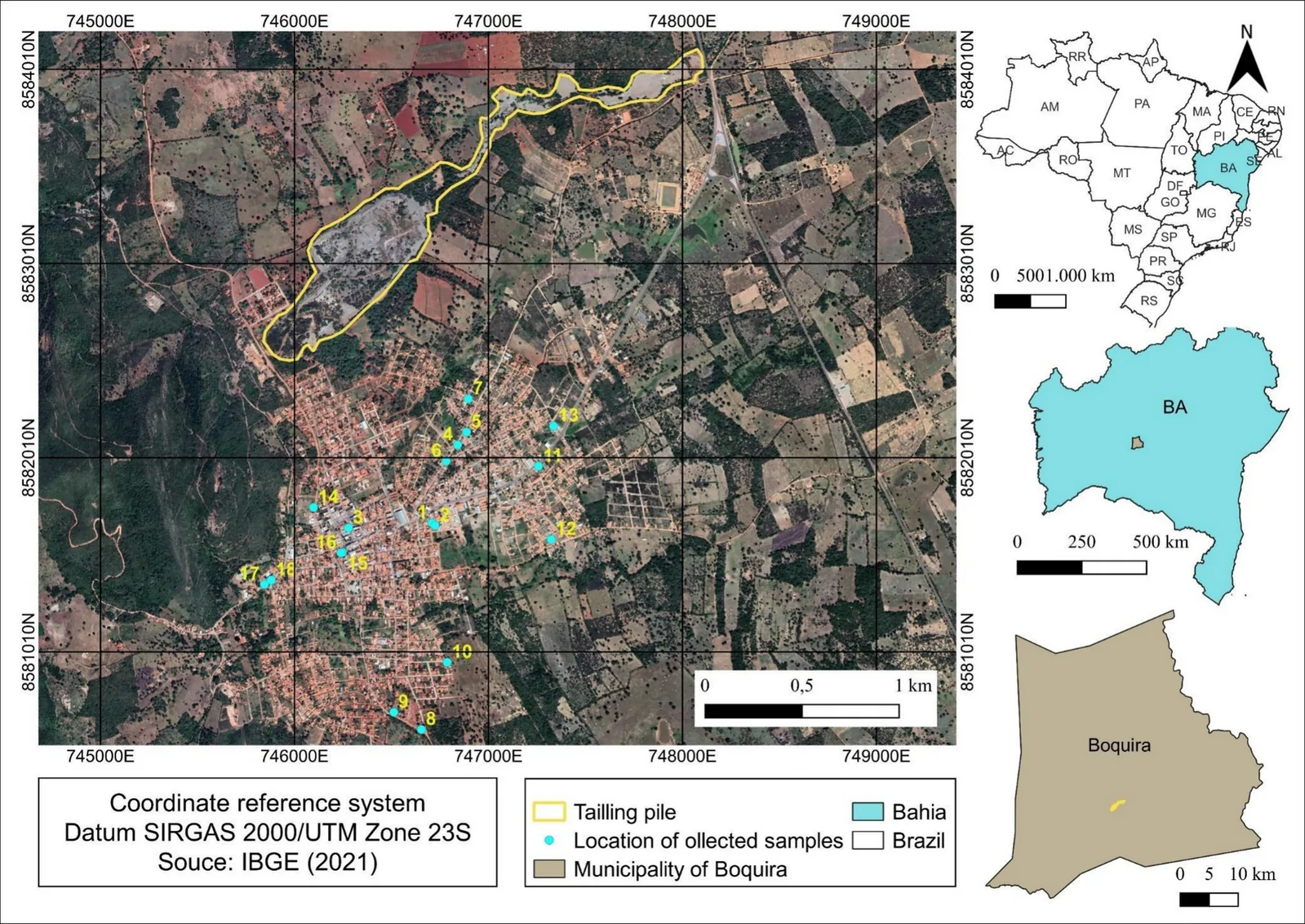
Location of the study area and spatial distribution of dust sampling points in Boquira. The map shows the urban area with the positions of the collected samples (blue points) and the outline of the abandoned tailings pile (yellow polygon)

The municipality of Boquira, situated in the south-central region of the state of Bahia, is approximately 669 km from the state capital, Salvador. The area previously housed a Pb-Zn processing plant for over three decades (Fig. 1). Geologically, the region is part of the Boquira Formation, a stratigraphic framework characterized by significant Pb and Zn mineralization due to the massive sulfide orebodies (Carvalho et al., 1982). This unit comprises an ancient gneissic-migmatitic complex (the Paramirim gneiss), the Boquira formation with volcanic-sedimentary rocks, metasedimentary rocks , especially quartzites of the Santo Onofre group, gabbros and granites and the Phanerozoic cover (Carvalho et al., 1982; Gomes et al., 2020).

Currently, the municipality contains an abandoned tailings pile covering approximately 3300 m^2^ and comprising an estimated 894,000 m^3^ of mining waste. This material has remained exposed for over 30 years near the urban area, posing risks to environmental quality and public health (dos Santos et al., 2020a, b). Dust samples, defined as fine particulate material deposited on soil surfaces or as thin layers on objects, were collected using a bristle brush and a plastic scoop, and stored in sterilized acrylic containers. A total of 18 strategically distributed sampling points were selected across the urban area, including residential water tank lids, benches, and tents at an open-air market, storage boxes at the water treatment facility, windows, commercial buildings, schools, and vehicle surfaces. The granulometric composition of the tailings material was determined using the pipette method (Gee & Bauder, 1986), yielding a predominantly sandy texture: 79.1% sand, 17.1% silt, and 3.8% clay.

### PTE levels in dust samples

The analyzed PTEs, including Pb, Zn, Cd, Cu, Mn, Fe, and Al, were selected based on previous studies conducted in the area (Paes et al., 2022). PTE concentrations in the dust samples were determined following USEPA Method 3051A (USEPA, 2007). Samples were oven-dried at 40 °C for 24 h, gently macerated, and sieved through a 200-mesh sieve. Approximately 0.5 g of each sample was weighed using an analytical balance (0.0001 g precision) and subjected to microwave-assisted digestion using 9 mL of concentrated HNO₃ and 3 mL of concentrated HCl. After digestion, the extracts were transferred to previously cleaned polyethylene bottles, cooled, and analyzed by inductively coupled plasma optical emission spectrometry (ICP-OES; PerkinElmer Optima 8300).

Because USEPA Method 3051A provides pseudo-total concentrations and does not fully dissolve silicate minerals, complementary analyses were conducted using energy-dispersive X-ray fluorescence spectrometry (EDX-8000). This approach enabled a more comprehensive assessment of the solid-phase elemental composition, particularly for elements associated with silicate structures, which are not completely recovered by acid digestion alone (MacLean & Barrett, 1993). One dust sample was excluded from the XRF analysis due to insufficient solid mass, so both pseudo-total and total elemental contents were evaluated. Pseudo-total concentrations obtained by USEPA Method 3051A were used to characterize the acid-extractable contaminant pool and for comparison with Brazilian soil quality reference values established by CONAMA Resolution No 420/2009 (Brasil, 2009). In contrast, total elemental contents obtained by XRF were used for geochemical ratio interpretation and source assessment. Importantly, USEPA Method 3051A was not used to infer bioavailability or bioaccessibility; oral bioaccessibility was evaluated independently using the 0.43 mol L^–1^ HNO_3_ extraction procedure described in the “Oral bioaccessibility” section. We acknowledge that PTE bioavailability depends on several interacting factors, including chemical speciation, mineralogical association, particle size, pH, redox conditions, organic matter, and physiological exposure conditions. Therefore, the bioaccessibility values reported here should be interpreted as operational estimates of the fraction soluble under simulated gastric-like conditions, rather than direct measurements of the bioavailability or actual human absorption (Rodriguez et al., 1999). More advanced approaches, including synchrotron-based techniques, would be necessary to determine PTE speciation and mineral hosts in greater detail.

Elemental-ratio tracers were also used to support the identification of potential dust sources. This approach is widely applied in geochemical studies of atmospheric dust, as relative enrichments or depletions of reactive elements can provide insight into source areas and transport pathways (Torghabeh et al., 2020; Yigiterhan et al., 2018). Accordingly, geochemical ratios were calculated for dust samples collected at selected points in Boquira and compared with those from the surface layer of a soil profile adjacent to the tailings pile, which served as a potential dust-source reference.

### Pollution indices and ecological risk assessment

To further assess Zn and Pb occurrence and environmental significance of in urban dust from Boquira, a set of widely applied pollution indices was employed: the geoaccumulation index (I_geo_), contamination factor (CF), enrichment factor (EF), and the potential ecological risk index (ER). These indices were calculated exclusively for Pb and Zn, given their direct association with local mining activities and their recognized toxicity.

These indices provide complementary insights into the degree of anthropogenic influence and the potential ecological risks posed by the PTEs. All calculations were based on local geochemical background concentrations representative of the study area. Background values were derived using multiple linear regression models developed from uncontaminated soil profiles associated with the two predominant geological units mapped in the region: the Boquira Formation, undivided banded iron formation association (PP12bfi), and the Boquira Granite, characterized as a medium- to coarse-grained biotite monzogranite (PP23gb) (see Topic S1).

The regression models utilize Mn, Fe, and Al concentrations as predictors to estimate baseline levels of Pb and Zn. This approach provides a more robust representation of natural geochemical variability, particularly in contexts dominated by sandy, quartz-rich materials, where elemental dilution and mineralogical controls are significant. By accounting for these factors, the method improves the discrimination between lithogenic contributions and anthropogenic enrichment. Background concentrations were estimated using Eqs. (1) and (2):

1 $${\lbrack Pb\rbrack}_{estimated}\:=\:a\:+\:b\lbrack Mn\rbrack\:+\:c\lbrack Fe\rbrack\:+\:d\lbrack Al\rbrack$$

2 $${\lbrack Zn\rbrack}_{estimated}\:=\:a\:+\:b\lbrack Mn\rbrack\:+\:c\lbrack Fe\rbrack\:+\:d\lbrack Al\rbrack$$

#### Geoaccumulation index (I_geo_)

The geoaccumulation index (I_geo_), originally proposed by Müller (1969), assesses the degree of metal accumulation relative to background concentrations. It is defined as:

3 $${I}_{geo}={ log}_{2}\left(\frac{\left[M\right]}{1.5 * [M{]}_{BG}}\right)$$

where [M] is the measured concentration of the element in the sample, and [M]_BG_ is the corresponding geochemical background value. The constant factor 1.5 is introduced to account for natural variability in background concentrations due to lithogenic effects and minor anthropogenic influences.

Based on the site-specific background values estimated via multiple linear regression, the I_geo_ values were interpreted according to Müller’s classification scheme: < 0 (unpolluted); 0–1 (unpolluted to moderately polluted); 1–2 (moderately polluted); 2–3 (moderately to heavily polluted); 3–4 (heavily polluted); 4–5 (heavily to extremely polluted); and > 5 (extremely polluted).

#### Contamination factor (Cf)

The contamination factor (CF) provides a direct measure of the contamination level by comparing an element's concentration in dust to its corresponding background value:

4 $$CF = \frac{\left[M\right]}{{\left[M\right]}_{BG}}$$

where [M] is the concentration of the element in the sample, and [M]_BG_ is its background concentration. CF values were interpreted as: CF < 1.5 (nil to very low contamination); 1.5 ≤ CF < 2 (low); 2 ≤ CF < 4 (moderate); 4 ≤ CF < 8 (high); 8 ≤ CF < 16 (very high); 16 ≤ CF < 32 (extremely high); and CF ≥ 32 (ultra-high).

#### Potential ecological risk index (ER)

The potential ecological risk index (ER), proposed by Hakanson (1980), integrates the ecological risk factors of individual elements and links their environmental effects with toxicological characteristics :

5 $$ER\:=\:TR\;\ast\;CF$$

6 $$RI\:=\:\sum\;ER$$

where ER is the ecological risk factor of a single element, TR is the toxic response coefficient, and CF is the contamination factor. The toxic response coefficients used were: Cr (2), Pb (5), Cd (30), Hg (40), As (10), Cu (5), Zn (1), and Ni (3). Risk levels for individual elements (ER) are classified as: ER < 40 (low risk); 40 ≤ ER < 80 (moderate); 80 ≤ ER < 160 (considerable); 160 ≤ ER < 320 (high); and ER ≥ 320 (very high). The integrated index (RI) is classified as: RI < 150 (low risk); 150 ≤ RI < 300 (moderate); 300 ≤ RI < 600 (considerable); and RI ≥ 600 (high).

#### Enrichment factor (ER)

The enrichment factor (EF) was used to evaluate the degree of anthropogenic influence by normalizing element concentrations against a conservative reference element. Iron (Fe) was selected due to its predominantly geogenic origin and relatively uniform distribution:

7 $$EF = \frac{\left([M]/[Fe]\right)}{\left([Fe{]}_{QRV}\right)}$$

where [M] and [Fe] represent the concentrations of the target element and Fe, respectively. The denominator utilizes the quality reference values (QRVs) obtained from reference soil profiles developed on the PP12bfi and PP23 geological units. These reference soils reflect natural conditions devoid of mining influence. While background values ([M]_BG_) used for I_geo_, CF, and RI were estimated via regression models, the QRV dataset was used exclusively for EF calculations. EF values were interpreted as: EF ≤ 2 (no enrichment or minimal enrichment); 2–5 (moderate); 5–20 (significant); 20–40 (very high); and ≥ 40 (extremely high enrichment).

### Oral bioaccessibility

The oral bioaccessibility of PTEs was assessed using a simplified extraction procedure previously tested by Rodrigues et al. (2018) and based on International Organization for Standardization (ISO/TS 17586: 2016) (ISO, 2016). This approach uses diluted HNO_3_ (0.43 mol L^–1^) to simulate gastric conditions and provides a practical alternative to the Unified BARGE Method (UBM), developed by the Bioaccessibility Research Group of Europe (BARGE; Wragg et al., 2011).

Briefly, approximately 1.00 ± 0.0001 g of dust sample was weighed into 50 mL centrifuge tubes using an analytical balance with 0.0001 g precision. Then, 10 mL of 0.43 mol L^–1^ HNO_3_ was added, and the suspension was shaken for 2 h in a horizontal shaker. After extraction, the suspension was filtered through slow-flow filter paper, and the extracts were stored at < 4 °C until analysis. The test was performed using the ≤ 250 µm dust fraction, as this particle-size range is considered representative of particles commonly ingested by children (USEPA, 2012). Finer fractions were not analyzed due to infrastructure limitations.

The concentrations of oral bioaccessible PTEs were determined by flame atomic absorption spectrometry (FAAS; AA240FS, Agilent Technologies®, USA). In vitro bioaccessibility (BA) was calculated as the ratio between the extractable PTE concentration in the oral or pulmonary phase and the total PTE concentration in the ingested dust, expressed as a percentage (Eq. 8; Oomen et al., 2002; Kang et al., 2011):

8 $$BA \left(\%\right) =\frac{CBA}{\left({C}_{T}\right)}\times 100$$

where BA is the bioaccessibility (%), C_BA_ is the bioaccessible concentration (mg kg^–1^), and C_T_ is the total PTE concentration determined via USEPA Method 3051A (mg kg^–1^).

### Human health risk assessment

#### Exposure risk assessment

Only Pb, Cd, Cu, and Zn were considered for health risk assessment across two exposure pathways (oral ingestion and dermal contact) and two population groups: children (0–18 years) and adults (> 18 years). Inhalation exposure was excluded, as it requires particles < 10 µm. The average daily doses (ADD) of PTEs were calculated using standard USEPA (1989):

9 $${ADD}_{Oral} = \frac{{C}_{S} \times IR \times ED \times EF }{BW \times AT} \times {10}^{-6}$$

10 $${ADD}_{Dermal }= \frac{{C}_{S} \times SA \times AF \times EF \times ABS}{BW \times AT} \times {10}^{-6}$$

where ADDₒᵣₐₗ and ADD_dₑᵣₘₐₗ are the average daily doses of PTEs via ingestion and dermal contact, respectively (mg kg^–1^ body weight day^–1^); Cₛ is the concentration of PTEs in dust (mg kg^–1^) determined using the U.S. EPA 3051 A method; IR is the dust ingestion rate (mg day⁻^1^); ED is the exposure duration (years); EF is the exposure frequency (days year^–1^); BW is the body weight of the exposed individual (kg); SA is skin-surface area (cm^2^), AF is the skin adherence factor (mg cm^–2^ h^–1^); ABS is the dermal absorption factor (unitless); AT is the averaging time (days); ET is the exposure time (h day^–1^); and PEF is the particle emission factor (m^3^ kg^–1^). Detailed parameter values used in the ADD calculations are provided in Table S3.

#### Carcinogenic and non-carcinogenic risk assessment

The non-carcinogenic risk was assessed using the hazard quotient (HQ), calculated as the ratio of the ADD to the corresponding reference dose (RfD) (Eqs. 11 and 12). The hazard index (HI), representing cumulative risk, is the sum of individual HQs across pathways (Eq. 13) (USEPA, 1989; Fernández-Caliani et al., 2019). While the USEPA does not currently establish an RfD for Pb (USEPA, 2025), an established literature value was adopted for HQ estimation (Chen et al., 2016).

11 $$HQoral = \frac{ADDoral}{RfD}$$

12 $$HQdermal =\frac{ADDdermal}{RfD}$$

13 $$HI ={\sum}_{i=1}^{n}\sum HQ$$

where HQ is the hazard quotient (unitless); RfD is the reference dose for each PTE (n) (mg kg⁻^1^ day⁻^1^); and the hazard index (HI) corresponds to the sum of the individual hazard quotients, representing the cumulative non-carcinogenic risk across elements and exposure pathways.

Furthermore, carcinogenic risk (CR), the probability of developing cancer over a lifetime, was estimated for probable human carcinogens (Pb and Cd) using Eq. 14 (Kan et al., 2021):

14 CR=ADD×SF

where SF is the slope factor (mg kg⁻^1^ day⁻^1^). Reference doses (RfD) for Pb, Zn, Cu, and Cd, and slope factors (SF) for Pb were adopted from Chen et al. (2016), USEPA (2012), Adimalla (2020), and Xiao et al. (2020). Although the official USEPA IRIS database does not establish an oral SF for Cd, classifying its carcinogenicity primarily through the inhalation pathway, this study adopted an alternative SF value to account for potential cumulative ingestion risks. This approach is consistent with recent literature evaluating ingestion exposure (Sidhu et al., 2026; Wang et al., 2026).

### Quality assurance and control

All glassware and plasticware were soaked in a 10% HNO_3_ acid bath for at least 24 h and rinsed with ultrapure water (18.2 MΩ·cm at 25 °C; Millipore Direct-Q system). Ultrapure water was utilized for all solutions. Extractions were performed in triplicate, and results are reported as the mean of the three replicates. All reagents were of analytical grade (Merck or Sigma-Aldrich). Method accuracy for acid digestion (USEPA 3051A) was verified using certified reference material SS-1 Contaminated Soil (EnviroMAT™).

### Statistical analysis

Statistical analyses were performed in R using the *easyANOVA* package (Arnhold, 2013). When data did not meet the assumption of normality, the upper limit of the 95% confidence interval (95th percentile) was used to represent the maximum reasonable exposure (Cao et al., 2014; Behrooz et al., 2021). To identify potential contamination sources, an unsupervised K-means clustering approach was applied to group sampling points with similar characteristics. The optimal number of clusters was determined by combining the elbow method and silhouette coefficient analysis. Prior to clustering, the PTE variables were standardized to eliminate the influence of differing measurement scales.

## Results

### Concentration and distribution of PTE in dust

The concentrations of PTEs, along with descriptive statistics and the corresponding limits of detection (LOD) and quantification (LOQ), are presented in Table 1. Mean concentrations followed the decreasing order: Fe > Al > Zn > Pb > Mn > Cu > Cd. Coefficients of variation ranged from 44.36 to 127.97%, with the lowest variability observed for Cu and the highest for Pb, followed by Cd (81.88%). This high variability suggests spatial heterogeneity, likely reflecting localized Pb enrichment in specific areas. Elemental concentrations for individual samples are detailed in Tables S2 and S4. Moreover, PTE concentrations ranged from 7.37 to 836.29 mg kg^–1^ for Pb, 42.36 to 866.43 mg kg^–1^ for Zn, 7.90 to 74.92 mg kg^–1^ for Cu, 142.84 to 824.38 mg kg^–1^ for Mn, 8,158.35 to 82,331 mg kg^–1^ for Fe, and 5,917.86to 48,519.66 mg kg^–1^ for Al. Cd concentrations remained below 50 mg kg^–1^ across all samples.

**Table 1:** Descriptive statistics of PTE concentrations (mg kg^–1^).

Statistic	Pb	Zn	Cd	Cu	Mn	Fe	Al
Minimum	7.37	42.36	4.26	7.90	142.84	8,158.35	5,917.86
Mean	166.25	236.76	8.66	43.03	383.14	45,239.72	21,076.11
Maximum	836.29	866.43	36.15	74.92	824.38	82,331.42	48,519.66
Median	94.16	213.07	6.19	35.34	348.93	41,880.09	19,537.57
SD	212.75	190.98	7.09	19.09	171.32	20,862.39	10,236.97
CV (%)	127.97	80.66	81.88	44.36	44.71	46.12	48.57
LOD	3.15 × 10^–6^	1.31 × 10^–6^	1.04 × 10^–6^	6.18 × 10^–7^	2.53 × 10^–8^	3.22 × 10^–7^	3.88 × 10^–6^
LOQ	1.05 × 10^–5^	4.38 × 10^–6^	3.45 × 10^–6^	2.06 × 10^–6^	8.44 × 10^–8^	1.07 × 10^–6^	1.29 × 10^–5^

*SD* standard deviation, *CV* coefficient of variation, *LOD* limits of detection, *LOQ* limits of quantification.

**Fig. 2:**
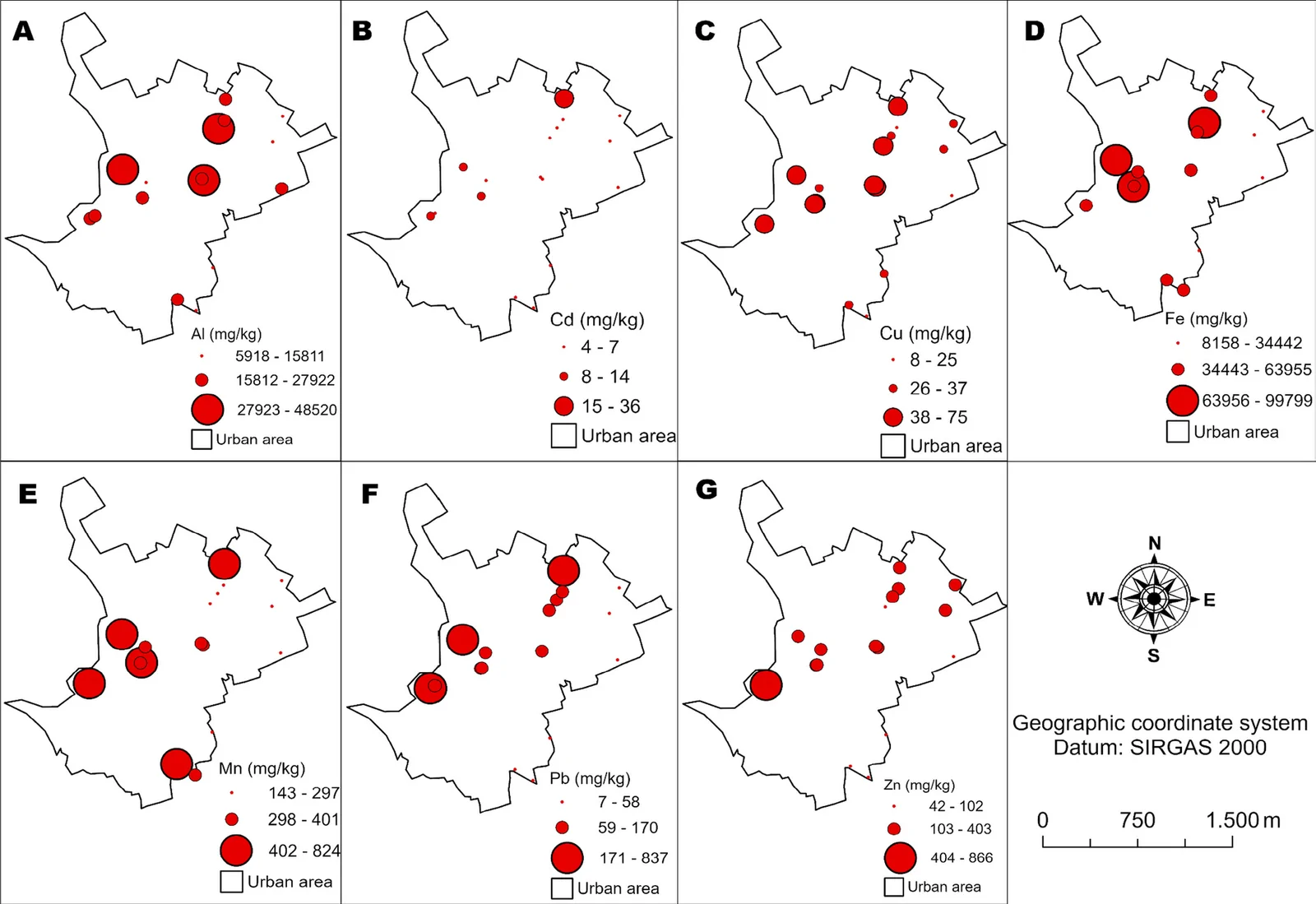
Spatial distribution of PTE concentrations in dust across the urban area of the municipality of Boquira, Bahia, Brazil: (**A)** Al, (**B)** Cd, (**C)** Cu, (**D)** Fe, (**E)** Mn, (**F)** Pb, and (**G)** Zn (mg kg^–1^). Symbol sizes are proportional to concentration ranges, as indicated in each panel. The urban area boundary is shown for reference.

The spatial distribution of PTE concentrations in urban dust, interpolated using inverse distance weighting (IDW) (Fig. 2), reveals pronounced heterogeneity across the urban area. Higher concentrations are predominantly observed in the northern sector and along the western extension of the city, including the northwest and southwest regions, particularly for Pb, Zn, Mn, and Cu. Although the tailings pile is located to the north of the urban area (Fig. 1), elevated concentrations in the western sector indicate that additional sources contribute to PTE enrichment in urban dust. Element-specific patterns further highlight these spatial contrasts. Al and Fe (Fig. 2A and D) show relatively widespread distributions with multiple zones of elevated concentrations, consistent with a dominant lithogenic control, although localized enrichment is also evident. In contrast, Cd (Fig. 2B) presents generally low concentrations with a few discrete hotspots, suggesting limited but spatially restricted inputs. Cu and Zn (Fig. 2C and G) display intermediate patterns, with scattered enrichment zones across the study area, particularly in the northern and western sectors. Pb (Fig. 2F) exhibits the highest spatial variability, with well-defined hotspots, corroborating the high coefficients of variation observed in the descriptive statistics and indicating localized contamination sources. Mn (Fig. 2E), although more evenly distributed, also shows areas of higher concentrations aligned with the western portion of the study area. Overall, the IDW-interpolated maps indicate that, while Al and Fe are largely controlled by geogenic sources, elements such as Pb, Zn, Cu, and Cd exhibit localized enrichment patterns, reflecting multiple, spatially variable anthropogenic inputs within the urban environment. To better understand the climatic conditions in the city of Boquira, data of relative humidity, wind direction, wind gust speed, and wind speed are shown in the supplementary material (Figure S3).

In addition to the clear spatial pattern of PTE concentrations, showing higher levels in the eastern sector of the urban area, the average geochemical ratios obtained for the collected dust (XRF analysis; Table S2) differed markedly from those measured in samples collected near the tailings pile. The dust samples exhibited the following ratios: Al_2_O_3_/TiO_2_ = 13, SiO_2_/TiO_2_ = 85, TiO_2_/Zr = 25, Zr/TiO_2_ = 0.05, and Rb/Sr = 0.74. In contrast, samples proximal to the tailings pile showed higher or distinct ratios: Al_2_O_3_/TiO_2_ = 20, SiO_2_/TiO_2_ = 96, TiO_2_/Zr = 67, Zr/TiO_2_ = 0.02, and Rb/Sr = 3.84. These differences indicate that the geochemical signature of urban dust is not solely controlled by the tailing material, suggesting the presence of multiple contamination sources within the study area (Table S6). This interpretation is further supported by cluster analysis, which identified three well-defined groups, consistent with distinct potential sources of PTE contamination (Figure S2).

### Pollution indices and ecological risk

**Fig. 3:**
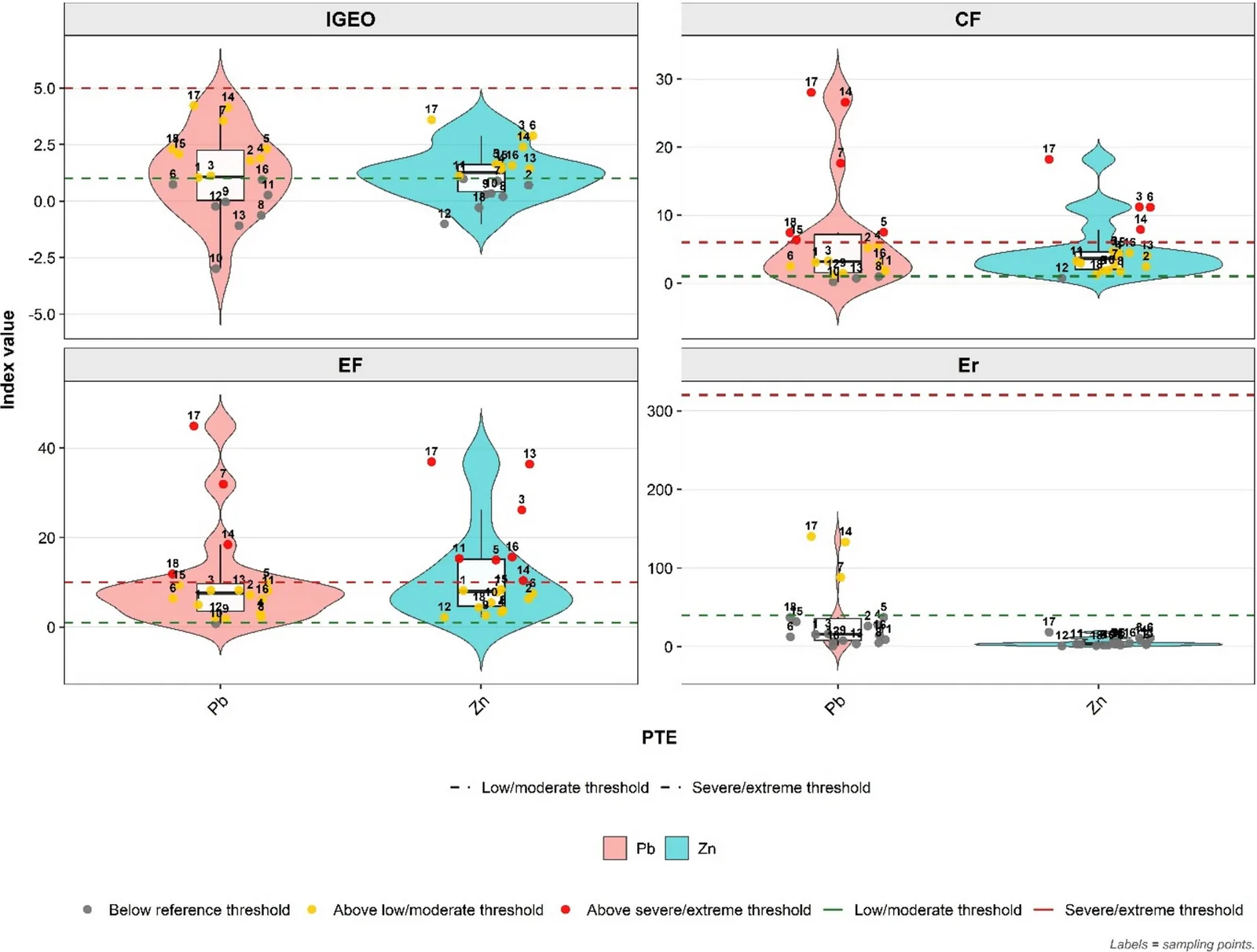
Geochemical indices for Pb and Zn, including geoaccumulation index (Igeo), contamination factor (CF), enrichment factor (EF), and ecological risk index (Er). Green dashed lines indicate thresholds corresponding to unpolluted, uncontaminated, or low-risk conditions, whereas red dashed lines represent severe contamination/pollution levels for Igeo, CF, and EF, and high ecological toxicity for Er

Despite the predominance of a quartz-rich sandy matrix, which would normally promote geochemical dilution of trace elements, Pb and Zn exhibited elevated geoaccumulation index (I_geo_) values, indicating substantial enrichment associated with mining-derived dust inputs in the urban area of Boquira (Fig. 3). The persistence of high contamination indices within a mineralogically diluted matrix demonstrates that anthropogenic contributions are sufficiently intense to override the dilution effect inherently imposed by quartz-dominated particles.

For Pb, 44% of the samples were classified as practically unpolluted (I_geo_ ≤ 0), whereas 39% ranged from moderately to heavily polluted (0 < I_geo_ ≤ 4), and 17% were classified with extreme pollution levels (Igeo > 4). The highest I_geo__Pb values were observed in samples 7 (3.6), 14 (4.1), and 17 (4.2), all located in close proximity to the mine tailings. These spatial trends indicate localized atmospheric deposition of tailings particles and hotspots from historical mining emissions. Enrichment near abandoned infrastructure suggests wind remobilizes contaminated particles, spreading toxic elements in the urban environment. In contrast, Zn showed a broader, more spatially diffuse contamination pattern. Approximately 55% of the samples were classified as moderately to heavily polluted, 39% as practically unpolluted, and only 6% as heavily polluted. This distribution indicates that Zn dispersion is less spatially restricted than Pb, likely reflecting differences in mineralogical occurrence, particle-size association, and atmospheric transport behavior.

The ecological risk index further emphasized the dominant environmental relevance of Pb contamination. For Pb, 66% of the samples were classified as presenting low ecological risk (Er < 40), whereas 17% showed moderate ecological risk (40 ≤ Er < 80) and 17% considerable ecological risk (80 ≤ Er < 160). The highest Er_Pb values were again recorded in samples 7 (88.0), 14 (132.8), and 17 (140.1), confirming that areas nearest to the historical pile remain active hotspots of ecological concern. These elevated ecological risk values demonstrate that, despite dilution within a largely inert quartz matrix, Pb concentrations remain sufficiently enriched to sustain potential adverse effects on urban ecosystems and human exposure pathways. For Zn, ecological risk was substantially lower, with all samples classified as low risk (Er < 40). This behavior is primarily related to the lower toxic-response coefficient assigned to Zn (Tr = 1), which reduces its relative ecological significance despite elevated contamination factors and enrichment levels in several locations. Consequently, although Zn exhibited widespread anthropogenic enrichment, its potential ecological hazard remained markedly lower than that associated with Pb.

In summary, the combined behavior of I_geo_, CF, EF, and Er unequivocally demonstrates that Pb is the primary ecotoxicological driver in the study area. More significantly, the persistence of elevated pollution indices for decades after mining cessation underscores the long-term environmental legacy of Pb–Zn exploitation in Boquira and highlights the ongoing role of atmospheric dust remobilization in sustaining urban contamination. These findings indicate that abandoned mining systems may persist as active sources of environmental exposure long after operational closure, particularly in semiarid environments where sparse vegetation cover and intense surface weathering facilitate continuous particulate redistribution.

### Oral bioaccessibility of PTEs

**Fig. 4:**
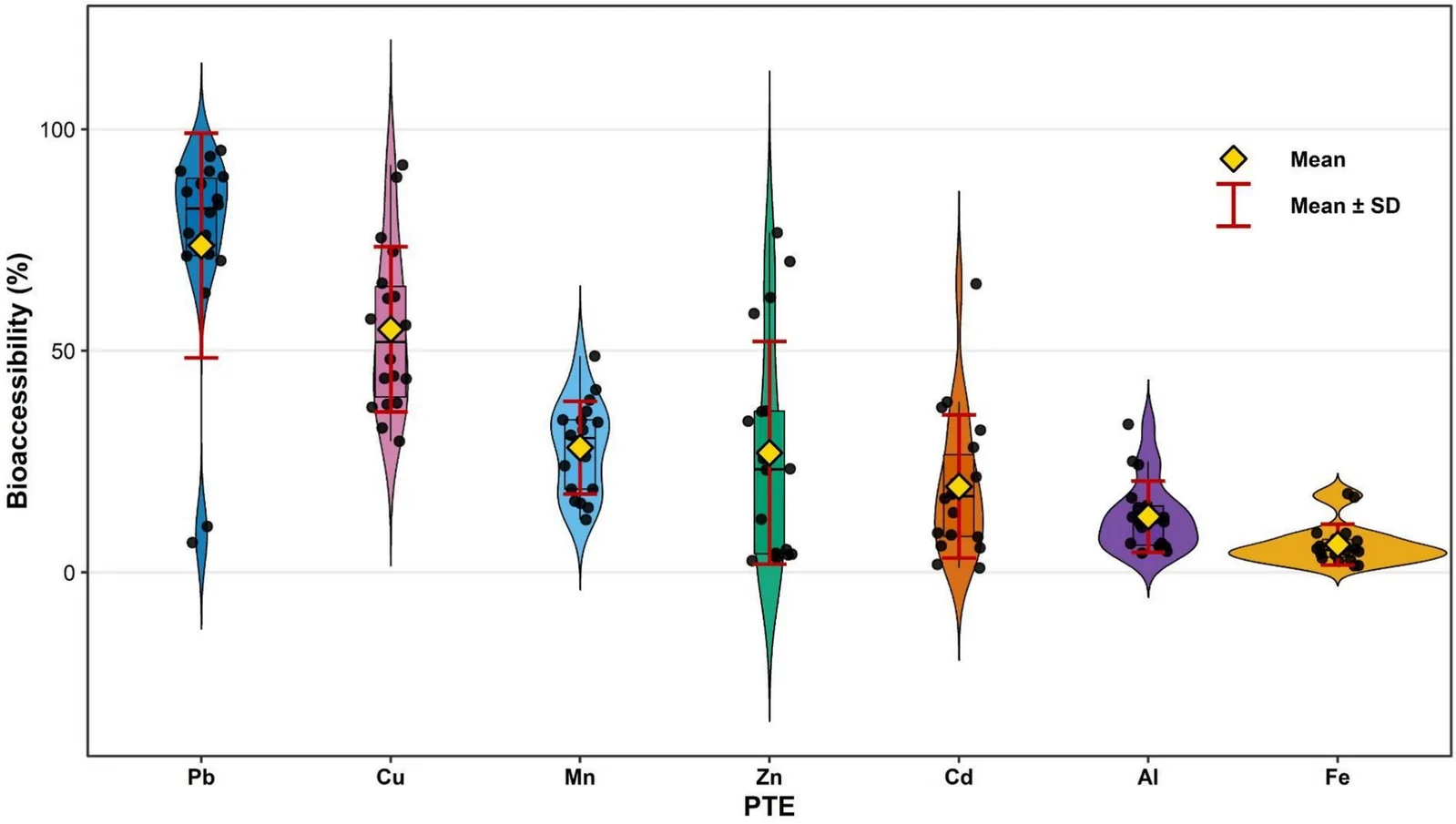
Oral bioaccessibility (%) of PTEs extracted using 0.43 mol L^–1^ HNO_3_.

Figure 4 presents oral bioaccessibility results for PTEs in urban dust from the Boquira municipality, Bahia State. Violin plots show pronounced inter-element variability in bioaccessible fractions, reflecting differences in mineralogy, reactivity, and contamination sources. Pb had the highest mean bioaccessibility (~ 75%), suggesting that Pb occurs in highly labile phases that readily solubilize under simulated gastric conditions, indicating potential gastrointestinal absorption. Cu had the second-highest mean bioaccessibility percentage (54.83%), followed by Mn (28.14%) and Zn (26.98%), both with intermediate values and considerable variability. Cd had a moderate mean bioaccessibility (19.36%) but a positively skewed distribution, suggesting spatial heterogeneity and localized enrichment. Fe (14.59%) and Al (12.58%) had the lowest mean bioaccessibility, with most observations at low levels, reflecting their association with less-soluble mineral phases under acidic conditions.

Despite low mean bioaccessibility, Fe had the widest distribution among PTEs, indicating substantial heterogeneity in reactive Fe-bearing minerals. This likely reflects mineralogical composition, weathering, and the coexistence of crystalline and poorly ordered Fe phases in urban dust. Pb and Cu are mainly associated with reactive phases, while Fe and Al are bound to stable lithogenic fractions. The broad distributions and outliers suggest strong spatial heterogeneity in contaminant sources and mineralogy. Total concentrations alone are insufficient to assess exposure risk, as elements with moderate concentrations may still pose significant toxicity when associated with highly bioaccessible fractions. The complete dataset is in Table S5.

#### Risk characterization

A human health risk assessment evaluated oral ingestion and dermal contact as primary exposure pathways for PTE-contaminated urban dust in adults and children. The assessment estimated non-carcinogenic risk using hazard quotient (HQ) and hazard index (HI), and carcinogenic risk (CR) for Pb and Cd. Oral ingestion was the dominant exposure pathway, accounting for more of the total risk than dermal contact. Children had higher exposure and risk values than adults. Pb was the main contributor to potential adverse health effects, especially for children exposed to incidental dust ingestion. While most evaluated elements had HQ values below the safety threshold, cumulative effects from multiple exposure pathways and elements increased total risk levels, especially for the youngest population.

#### Hazard quotient (HQ) and carcinogenic risk

Table 2 presents the hazard quotients (HQs) for Pb, Zn, Cd, and Cu, calculated for oral ingestion and dermal contact pathways in both adults and children. Overall, oral exposure produced substantially higher HQ values than dermal exposure for all evaluated elements. Among the analyzed PTEs, Pb showed the highest non-carcinogenic risk, with HQ values exceeding the reference threshold (HQ > 1) for oral exposure in children (HQ = 2.04), indicating the potential occurrence of adverse health effects associated with chronic exposure. Cd also presented relatively elevated HQ values for children via ingestion (HQ = 2.20 × 10^–1^), although remaining below the critical threshold.

**Table 2:** Hazard quotient (HQ) values estimated for Pb, Zn, Cd, and Cu through oral ingestion and dermal contact pathways for adults and children

Hazard quotient (HQ)				
	Oral		Dermal	
	Adult	Children	Adult	Children
Pb	2.18 × 10^–1^	2.04 × 10^0^	2.42 × 10^–3^	6.33 × 10^–2^
Zn	2.16 × 10^–3^	2.01 × 10^–2^	1.79 × 10^–5^	4.70 × 10^–4^
Cd	2.36 × 10^–2^	2.20 × 10^–1^	3.92 × 10^–3^	1.03 × 10^–1^
Cu	2.38 × 10^–3^	2.22 × 10^–2^	1.32 × 10^–5^	3.46 × 10^–4^

Dermal exposure contributed comparatively little to the total non-carcinogenic risk, with HQ values being several orders of magnitude lower than those estimated for oral exposure. For Zn and Cu, HQ values remained well below unity for both exposure pathways and population groups, indicating low non-carcinogenic risk under the evaluated exposure conditions. For all evaluated elements and exposure pathways, HQ values estimated for children were consistently higher than those calculated for adults. This pattern is associated with lower body weight and higher ingestion rates typically observed in children, increasing their relative exposure to contaminated dust. These results indicate that children are the most vulnerable population in the study area, particularly regarding Pb exposure from incidental dust ingestion.

#### Hazard index assessment (HI)

The hazard index (HI) values obtained for adults and children are presented in Table 3. Among the evaluated exposure pathways, oral ingestion accounted for the dominant contribution to total non-carcinogenic risk in both population groups, whereas dermal contact contributed comparatively little. Total HI values were substantially higher for children (2.47 × 10⁰) than for adults (2.53 × 10⁻^1^), indicating greater susceptibility of children to adverse non-carcinogenic effects associated with exposure to contaminated urban dust.

**Table 3:** Average hazard index (HI) values for adults and children, considering oral, dermal, and inhalation exposure routes, along with the total HI for each group. The carcinogenic risk (CR) for Pb and Cd is also presented

Exposure routes	Adults	Children		
Oral (Σ PTE)	2.46 × 10^–1^	2.30 × 10^0^		
Dermal (Σ PTE)	6.37 × 10^–3^	1.67 × 10^–1^		
Oral + dermal HI (Σ PTE)	2.53 × 10^–1^	2.47 × 10^0^		
Pb (all exposure routes)	2.21 × 10^–1^	2.10 × 10^0^		
Zn (all exposure routes)	2.18 × 10^–3^	2.06 × 10^–2^		
Cd (all exposure routes)	2.76 × 10^–2^	3.23 × 10^–1^		
Cu (all exposure routes)	2.40 × 10^–3^	2.26 × 10^–2^		
Carcinogenic risk				
	Oral		Dermal	
	Adult	Children	Adult	Children
Pb	6.49 × 10^–6^	6.06 × 10^–5^	NA	NA
Cd	1.44 × 10^–4^	1.34 × 10^–3^	2.39 × 10^–7^	6.26 × 10^–6^
∑CR*	1.50 × 10^–4^	1.40 × 10^–3^	2.39 × 10^–7^	6.26 × 10^–6^

*NA *not applicable. *The sum of CR

Regarding carcinogenic risk, Cd showed the highest CR values among the evaluated elements, especially through oral exposure. Carcinogenic risks associated with Cd ingestion exceeded the acceptable range proposed by regulatory agencies (10^–6^–10^–4^) for both adults and children, with children presenting the highest risk values (1.34 × 10^–3^). In contrast, Pb exhibited lower CR values, remaining within or near the acceptable risk range. Dermal carcinogenic risk was negligible compared with oral exposure for both elements.

**Fig. 5:**
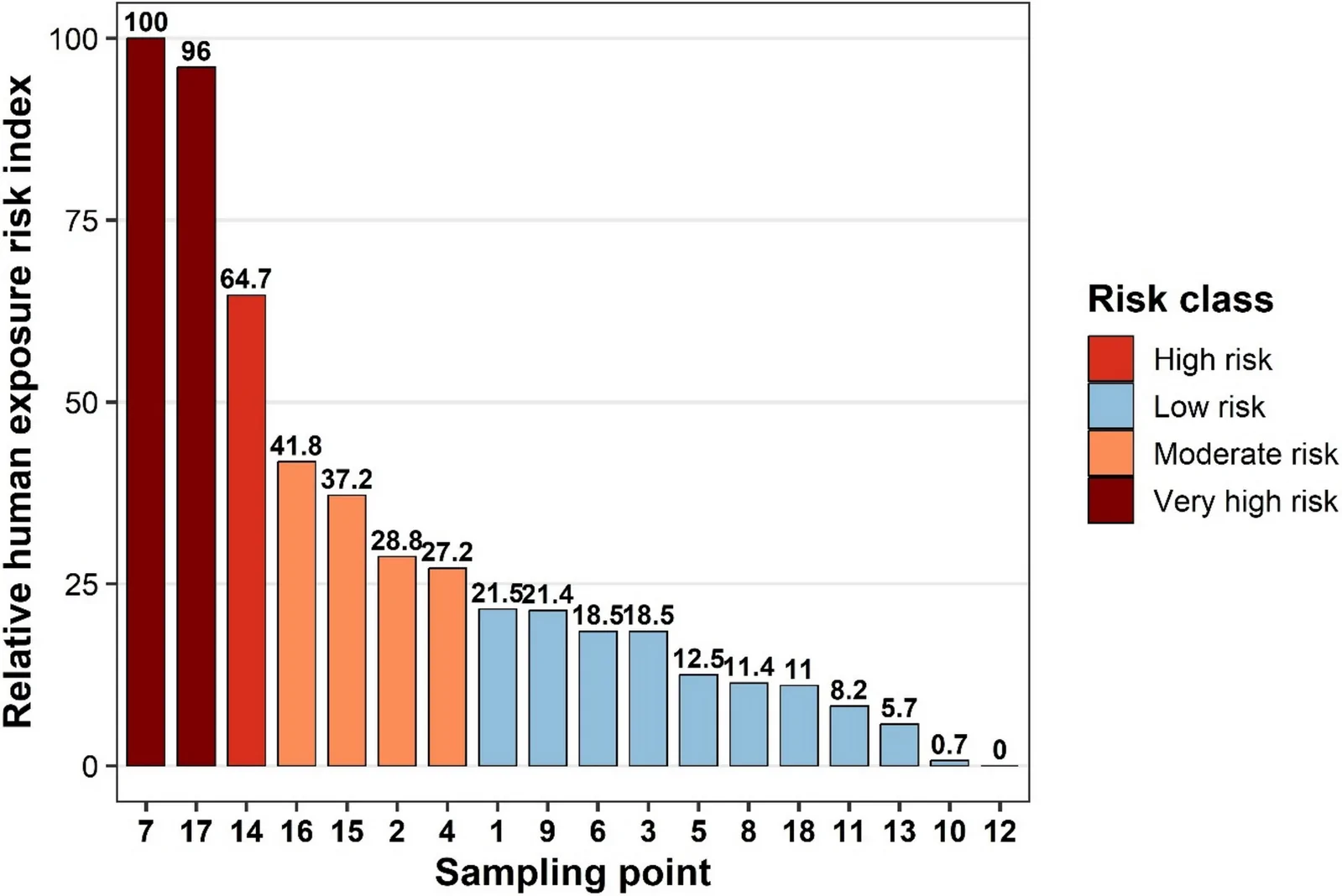
Dust sampling points and their relative human exposure risk

For both adults and children, the contribution of individual elements to total HI followed the decreasing order: Pb > Cd > Cu > Zn. Pb was the main contributor to total non-carcinogenic risk, accounting for approximately 85% of total HI. Furthermore, approximately 85.59% of Pb-related risk originated from the oral exposure pathway, reinforcing ingestion as the principal route associated with potential adverse health effects in the study area. The elevated HI values observed for children were primarily associated with Pb exposure, whose cumulative HI exceeded the safety threshold (HI > 1), suggesting potential risks for the development of non-carcinogenic health effects. In contrast, HI values for Zn and Cu remained substantially below critical levels for both adults and children. According to the carcinogenic risk classification proposed by Kan et al. (2021), CR values ranging from 10⁻⁶ to 10⁻^4^ are considered acceptable. In the present study, Cd ingestion was the only exposure condition exceeding this acceptable range for both adults and children, highlighting Cd as a critical concern for long-term carcinogenic effects. A relative ranking of the samples based on human health risk is shown in Fig. 5, which aims to assist in prioritizing remediation or intervention efforts.

## Discussion

### Distribution of PTEs in the urban area

The average PTE concentrations in urban dust were generally below the prevention and residential investigation thresholds established by CONAMA Resolution No. 420/2009 (Brasil, 2009), except for Pb and Cd, whose mean concentrations exceeded the residential regulatory limits. Rather than representing isolated contamination hotspots, these elevated concentrations reflect the persistence of legacy mining contamination within the urban landscape decades after mine closure. In post-mining environments, abandoned tailings deposits frequently evolve into long-term secondary sources of atmospheric contamination, continuously supplying fine particles to surrounding ecosystems through erosion and resuspension processes (Cheng et al., 2020; Gao et al., 2023; Santos et al., 2023; Zhong et al., 2020).

The combined spatial, geochemical, and clustering evidence suggests that the Boquira tailings pile serves as the primary source of Pb- and Zn-enriched particulates dispersed throughout the urban area. Due to the tailings material’s composition predominantly of sand-sized particles, its low structural stability and limited vegetation cover facilitate continuous aeolian remobilization, particularly during the dry season when strong winds intensify dust transport (Santos et al., 2020a, b). Under semi-arid conditions, repeated cycles of erosion, deposition, and resuspension transform mine waste into an active atmospheric contamination source rather than a static depositional feature**,** and in this semi-arid climate, sand-sized dust particles are widely documented (Yu et al., 2025), especially the fine-sand sized (Mesquita et al., 2024).

However, the distinct geochemical signatures observed between urban dust and tailings material suggest that contamination is not solely controlled by direct tailings inputs. The divergence in elemental ratios, coupled with the clustering of sampling points, indicates mixing between mine-derived particulates and locally derived materials. In this context, former extraction zones and secondary waste-accumulation sites located along the western mountainous sector are likely to contribute additional anthropogenic inputs. Consequently, urban dust composition appears to reflect the interaction between legacy mining emissions, local geomorphological controls, and urban sediment redistribution processes. By capturing these complex interactions, unsupervised clustering methods prove to be powerful tools for isolating mixed contamination sources in mining-impacted environments, as previously demonstrated by Li et al. (2017) and Ferreira et al. (2019).

The spatial organization of contamination further suggests that atmospheric transport dynamics significantly influence the patterns of PTE accumulation. The geomorphological characteristics of the Paramirim Valley likely facilitate the preferential retention of contaminated particulates through valley-mountain circulation dynamics. This topographic barrier effect may account for the recurrent enrichment observed in western residential areas, where suspended particles transported from the mining pile site are likely trapped and redeposited. Consequently, contaminant accumulation is not solely determined by the distance from the tailings pile, but rather the result of coupled atmospheric and geomorphological processes that regulate particle mobility across the urban landscape.

The samples represent a diffuse reservoir of urban dust present in the city, rather than independent exposure environments. Categorizing samples according to surface type would not necessarily reflect distinct contamination sources or significantly different dust compositions, but primarily local differences in particle accumulation efficiency. The selected surfaces are used as passive samplers, and the risk assessment should be interpreted as an analysis of diffuse, rather than point-source, urban dust contamination. When considering urban dust contamination, it is not unusual to disregard the surface variation and consider other factors such as sampling accessibility, operational continuity, and proximity to residential areas to ensure relevance to human exposure scenarios (Ediagbonya et al., 2026).

Although PTE concentrations in urban dust were lower than those reported for soils and tailings from the same mining district (Paes et al., 2022, 2023a, b, c; Santos et al., 2020a, b), dust represents a substantially more dynamic and bioaccessible contamination pathway. In highly weathered tropical systems, Fe-, Al-, and Mn-rich oxides can effectively retain metals within the soil matrix through sorption processes (dos Santos et al., 2017; Gloaguen & Passe, 2017). Nevertheless, once incorporated into fine particulate matter, these contaminants become considerably more mobile and accessible for human exposure. This distinction highlights the importance of considering particle mobility and exposure pathways, rather than relying solely on total concentration in bulk environmental matrices.

The enrichment factors and contamination indices corroborate the predominance of anthropogenic Pb inputs. Elevated EF values calculated for Pb signify enrichment exceeding natural lithogenic variability, even in a geological setting naturally enriched in Fe- and Pb-bearing lithologies (Carvalho et al., 1982; Gomes et al., 2020). Notably, the selection of Fe as a normalization element may partially obscure anthropogenic enrichment in samples originating from Fe-rich parent materials. Consequently, the occurrence of high Pb enrichment despite elevated natural Fe concentrations strongly supports a mining-related origin associated with ore extraction and processing activities.

### Bioaccessibility

Among the evaluated elements, Pb exhibited the highest oral bioaccessibility, indicating that a substantial fraction of Pb associated with urban dust is highly labile. This behavior suggests that the toxicological relevance of Pb in Boquira is not solely determined by its concentration, but also by its chemical lability and physiological availability after ingestion. Consequently, relatively moderate total concentrations may still represent significant exposure risks when associated with highly soluble mineral phases (Kastury et al., 2017; Ren et al., 2020).

However, the high bioaccessibility percentages observed in specific samples (e.g., samples 12 and 17) can be ascribed to the overestimation of bioaccessible estimated by diluted nitric acid. One reason for it can be the lower pH of the chemical extraction compared to other bioaccessibility methods (Vasques et al., 2020), producing a 1.4-fold increase compared to other chemical procedures (Fernández-Caliani et al., 2019). Furthermore, because bioaccessibility percentages were calculated relative to the pseudo-total content extracted via USEPA Method 3051A, which does not completely dissolve residual silicate matrices, the calculated fractions may be mathematically higher than those relative to absolute total elemental contents.

The elevated Pb bioaccessibility likely reflects the predominance of secondary Pb carbonates, particularly hydrocerussite, previously identified in mine wastes from the region (Paes et al., 2025). These mineral phases are thermodynamically unstable under acidic conditions (Tai et al., 2013) and readily dissolve during chemical extraction, increasing the fraction potentially available for intestinal absorption. This mechanism helps explain why Pb bioaccessibility in urban dust remained high even though total concentrations were lower than those reported directly in mine tailings. Indeed, in a study within the same area, Paes et al. (2025) found that in the tailings themselves, bioaccessible contents of Pb were around 50% of the total contents. Although these operational variations must be acknowledged when comparing different extraction methodologies, our findings suggest that atmospheric transport and sediment mixing may dilute bulk elemental concentrations without proportionally reducing the highly bioaccessible fraction. Even knowing that simplified in vitro extraction methods do not directly measure in vivo bioavailability or actual biological uptake, they provide an experimentally based and operational estimate of the fraction of PTEs that may become soluble under gastric-like conditions. The 0.43 mol L⁻^1^ HNO₃ extraction used here has been previously tested and proposed as a practical alternative to more complex gastrointestinal protocols, including the Unified BARGE Method (UBM) (Li et al., 2015; Rodrigues et al.,^ 2018^; Pelfrêne et al.,  2020). Pb bioaccessibility is not controlled by pH alone since chemical speciation, mineralogical association, particle size, organic matter, redox conditions, and the formation of soluble ionic or hydrolyzed complexes also strongly influence Pb solubility and potential uptake. Therefore, the high Pb bioaccessibility observed in this study should be interpreted as an operational indication of Pb solubilization under simulated gastric conditions, rather than as a direct measurement of in vivo bioavailability. Further speciation analyses, including synchrotron-based techniques, would be necessary to confirm the specific Pb-bearing phases and soluble species controlling Pb mobility and bioaccessibility.

This distinction highlights an important limitation of conventional contamination assessments based exclusively on pseudo-total or total concentrations. The solution used here, 0.43 M HNO_3_, is traditionally employed to assess geochemically reactive elements in soil (Groenenberg et al., 2017) and is a proxy of environmental mobility. In post-mining systems, the environmental behavior and toxicological significance of PTEs are strongly dependent on mineralogical speciation and phase stability (Ettler et al., 2019; Pelfrêne et al., 2017). Therefore, bioaccessibility analyses provide a more realistic representation of exposure potential by simulating the chemical reactivity of contaminated particles. However, a key limitation is that only the potentially oral-bioaccessible fraction was estimated via this chemical proxy; furthermore, the heterogeneity of the sampled urban surfaces indicates that not all dust deposits will be equally accessible to direct human contact or ingestion pathways.

Zn, Cd, and Cu also exhibited moderate to high bioaccessibility values, suggesting that a considerable proportion of these elements is associated with reactive mineral fractions. This pattern is consistent with the possible presence of reactive fractions, such as acid-soluble phases, poorly crystalline oxides, or carbonate-associated metals, which may dissolve under acidic gastrointestinal-like conditions (Pelfrêne et al., 2017; Xie et al., 2023). However, the specific mineral phases controlling this behavior were not directly identified in this study, although confirmed by others (Paes et al., 2025). Therefore, this interpretation should be considered a plausible explanation rather than definitive evidence of the occurrence of these phases. The relatively high bioaccessibility of Cd is particularly concerning, as even low concentrations may contribute disproportionately to carcinogenic risk due to its high toxicological potential.

Particle-size distribution likely exerts additional control on oral bioaccessibility. Finer dust generally possesses higher specific surface area, greater metal enrichment, and enhanced chemical reactivity, increasing both environmental mobility and physiological solubility (Zhou et al., 2024). Because the present study evaluated particles ≤ 250 µm, the actual exposure potential associated with the finest, most ingestible fractions may be underestimated. Previous studies have shown that medium and fine fractions constitute the main carriers of Pb in urban dust systems (Chang and Li, 2020; Khademi et al., 2020).

### Health risk assessment

#### Non-carcinogenic risk

The predominant oral exposure as the primary contamination pathway reflects the strong interaction between contaminated urban dust and human behavioral exposure patterns, particularly in children. In semi-arid urban environments affected by mining residues, chronic dust resuspension increases the likelihood of incidental ingestion through hand-to-mouth activity, surface contact, and indoor accumulation of fine particles. Consequently, legacy mine waste becomes incorporated into the daily exposure environment of nearby populations.

The elevated hazard index values observed in children indicate that vulnerability is determined not only by environmental contamination levels but also by physiological susceptibility. Lower body mass, higher ingestion rates, and greater contact with ground-level environments substantially increase relative exposure during childhood, amplifying the toxicological significance of contaminated dust (Ahmad et al., 2019). In this context, children represent a disproportionately exposed population group in post-mining environments. Pb was the dominant contributor to total non-carcinogenic risk, indicating that Pb remains the principal toxicological driver of contamination in Boquira. Importantly, the exceedance of the hazard index (HI) threshold occurred even after atmospheric dilution of tailings-derived material within the urban environment. This demonstrates that relatively dispersed contamination may still sustain significant exposure risk when combined with high oral bioaccessibility and chronic resuspension dynamics. Similar exposure patterns have been widely reported in mining-impacted environments, where oral ingestion consistently represents the dominant exposure route (Gao et al., 2023; Gope et al., 2017; Xiao et al., 2020).

The relatively low contribution of dermal exposure underscores the significance of physicochemical interactions in determining contaminant transfer efficiency. While dermal contact may contribute to cumulative exposure, ingestion remains the predominant pathway due to gastrointestinal dissolution, which facilitates substantially greater metal mobilization and absorption compared to skin contact. Furthermore, the omission of an inhalation risk assessment represents an important limitation in scope. Considering the semi-arid conditions, strong seasonal winds, and continuous atmospheric resuspension observed in Boquira, inhalation of fine contaminated particulates may represent an additional relevant exposure route. Consequently, future investigations that integrate pulmonary bioaccessibility, atmospheric monitoring, and particle-size characterization are essential for a more comprehensive assessment of human exposure.

#### Carcinogenic risk

The carcinogenic risk assessment further underscores the long-term health implications of chronic exposure to contaminated urban dust. Cd represented the primary contributor to carcinogenic risk, particularly through oral ingestion, with CR values exceeding acceptable thresholds for both adults and children. These results indicate that even relatively low Cd concentrations may cause disproportionate toxicological impacts due to its high carcinogenic potency and cumulative behavior in biological systems (Kan et al., 2021). Moreover, the CR obtained in the present study was higher when compared to other regions of Brazil obtained by Lima et al. (2023) for dust samples collected in another urban area, the city of Recife (Northeast of Brazil). 

The predominance of oral carcinogenic risk underscores the critical role of bioaccessible dust ingestion as an exposure pathway in mining-impacted urban areas. Unlike direct occupational exposure scenarios, contamination in Boquira occurs through chronic low-dose environmental exposure, where repeated ingestion of contaminated particles over long periods may gradually increase carcinogenic burden within the exposed population. The lower calculated carcinogenic risk of Pb relative to Cd primarily reflects differences in their cancer SF, in addiiton to dilution during atmospheric transport and during urban sediment mixing. However, this apparent reduction in carcinogenic risk should not be interpreted as a reduction in environmental relevance, since Pb remained the dominant contributor to non-carcinogenic effects and exhibited the highest bioaccessible fraction among all evaluated elements.

More broadly, the results demonstrate that abandoned mine residues remain active sources of human exposure decades after mining cessation. In this sense, the Boquira case exemplifies how legacy mining contamination can persist through atmospheric redistribution pathways, transforming urban dust into a chronic vector of environmental exposure. Similar long-term contamination patterns have been reported in other mining-impacted regions, including Santo Amaro, Brazil (Amorim et al., 2021; Silva et al., 2017). These findings highlight the need for integrated monitoring strategies that combine geochemical characterization, bioaccessibility assessment, atmospheric transport analysis, and biomonitoring to better constrain long-term health risks in post-mining environments.

## Conclusions

This study shows urban dust in Boquira as a key human exposure route to PTEs from legacy Pb–Zn mining. While most mean concentrations are below Brazilian thresholds, Pb and Cd surpass safety and residential limits, showing ongoing contamination decades post-mine closure. Analyses indicate that abandoned tailings are the main source of Pb and Zn particles, with geomorphological and atmospheric processes influencing their dispersion across the city. However, mixed sources are also observed. Geochemical indices showed significant human enrichment, especially for Pb and Zn, with several sites severely polluted. Pb had the highest oral bioaccessibility, meaning that a high content is potentially dissolved under acidic solutions simulating stomach pH. Cd is the primary contributor to carcinogenic risk, with risk values above acceptable limits, especially for children. The main exposure pathway is chronic ingestion of contaminated dust.

Consequently, legacy mine tailings in Boquira remain environmentally active, continuing to redistribute bioaccessible PTEs into residential areas. Geochemical indices, bioaccessibility assessment, spatial analysis, and human health risk modeling are crucial for understanding contamination dynamics and exposure pathways in post-mining urban systems. Therefore, long-term environmental monitoring and mitigation strategies are needed in Boquira and in similar mining-impacted regions. Future studies should include atmospheric monitoring of finer particle-size fractions, pulmonary bioaccessibility, and biomonitoring to better constrain human exposure and health risks.

## Supplementary Information

Below is the link to the electronic supplementary material.

ESM 1 Supplementary Material 1 (DOCX 3.93 MB)

## Data Availability

Any other details of this study are available from the corresponding author upon reasonable request.
