# Supplementary material for: Legacy Pb–Zn mine tailings as persistent sources of bioaccessible potentially toxic elements in urban dust: geochemical signatures, exposure pathways, and human health risks
Source: Environ Monit Assess. 2026 Aug 13;198(9):942. doi: 10.1007/s10661-026-15743-x (PMC13473335; doi:10.1007/s10661-026-15743-x)
Supplement: Supplementary file 1 [file 10661_2026_15743_MOESM1_ESM.docx]

**Supplementary Information**

**Legacy Pb–Zn mine tailings as persistent sources of bioaccessible potentially toxic elements in urban dust: geochemical signatures, exposure pathways, and human health risks**

Edvaldo Renner da Costa Cardoso^a^, Rugana Imbaná^a^, Ésio de Castro Paes^a^, Isabela C. F. Vasques^a^, Oldair Vinhas Costa^b^, Thomas Vincent Gloagen^b^, Emanuelle Mercês Barros Soares^a^

^a^ Department of Soils, Federal University of Viçosa, R. Purdue S/N, Campus Universitário, Viçosa, Minas Gerais, 36570-900, Brazil.

^b^ Center for Agricultural, Environmental and Biological Sciences, Federal University of Recôncavo of Bahia, 44380-000, Cruz das Almas, Brazil

*Corresponding author: [isabela.filardi@ufv.br](mailto:isabela.filardi@ufv.br)

**Topic S1. Calculation of individual backgrounds for each dust sample:**

The estimation of background values from major elements using multiple regression offers several advantages: (i) it enables the determination of a specific background value for each sample, enhancing accuracy; (ii) it addresses the challenge of identifying reliable natural background levels in environments affected by mining activities; (iii) it helps to reduce uncertainty regarding the geogenic or anthropogenic origin of metals in the analyzed samples; and (iv) it accounts for the inherent variability and potential multiplicity of geological sources in aeolian dust samples.

[Pb]_estimated_	= a+ b[Mn] + c[Fe] + d[Al]

[Zn]_estimated_	= a+ b[Mn] + c[Fe] + d[Al]

- Estimation of Pb background values, using the reference uncontaminated samples of the soil profile developed on the Paleoproterozoic geologic units PP12Bfi (sedimentary formation with ferriferous formation) and PP23 (granites) (CPRM, 2014).

Table S1.1: Regression analysis for Pb determination of background values.

| R multiple | 0.989736357 |
| --- | --- |
| R square | 0.979578056 |
| R adjusted square | 0.96732489 |
| Standard error | 2.572097009 |
| Observations | 9 |

| Table S1.2: ANOVA analysis for estimation of Pb background values  ANOVA | | | | | |  |  |  |
| --- | --- | --- | --- | --- | --- | --- | --- | --- |
|  | **DF* | *SQ* | *MS* | *F* | *p* |  |  |  |
| Regression | 3 | 1586.7 | 528.9 | 79.94 | 0.0001 |  |  |  |
|  | 5 | 33.1 | 6.6 |  |  |  |  |  |
| Total | 8 | 1619.7 |  |  |  |  |  |  |
|  |  |  |  |  |  |  |  |  |
|  | *a b c d* | ***SE* | *t* | *valor-P* | *95% L.* | *95% U.* | *L. 95.0%* | *U. 95.0%* |
| Intersection | 37.538 | 11.733 | 3.199 | 0.024 | 7.377 | 67.698 | 7.377 | 67.698 |
| Mn | 1.02E-02 | 6.26E-03 | 1.626 | 1.65E-01 | -5.91E-03 | 2.63E-02 | -5.91E-03 | 2.63E-02 |
| Fe* | 6.57E-04 | 2.44E-04 | 2.693 | 4.32E-02 | 2.98E-05 | 1.28E-03 | 2.98E-05 | 1.28E-03 |
| Al* | -2.25E-03 | -2.25E-03 | -9.666 | 2.01E-04 | -2.85E-03 | -1.65E-03 | -2.85E-03 | -1.65E-03 |

* DF: Degree of freedom; SQ: sum of square; MS: mean sum of square.

** SE: standard error; L: lower; U: upper

Figure S1.1: Estimated versus real concentration of Pb in samples.

- Estimation of zinc background values, using the reference uncontaminated samples of the soil profile developed on the Paleoproterozoic geologic units PP12Bfi and PP23.

| R multiple | 0.981063 |
| --- | --- |
| R square | 0.962485 |
| R adjusted square | 0.939976 |
| Standard error | 7.182987 |
| Observations | 9 |

Table S1.3: Regression analysis for Zn determination of background values.

Table S1.4: ANOVA analysis for estimation of Zn background values

| ANOVA | | | | | |  |  |  |
| --- | --- | --- | --- | --- | --- | --- | --- | --- |
|  | **DF* | *SQ* | *MS* | *F* | *p* |  |  |  |
| Regression | 3 | 6618.60 | 2206.20 | 79.94 | 0.0001 |  |  |  |
|  | 5 | 257.98 | 51.60 |  |  |  |  |  |
| Total | 8 | 6876.58 |  |  |  |  |  |  |
|  |  |  |  |  |  |  |  |  |
|  | *a b c d* | ***SE* | *t* | *valor-P* | *95% L.* | *95% U.* | *L. 95.0%* | *U. 95.0%* |
| Intersection | 40.055 | 32.766 | 1.222 | 0.276 | -44.174 | 124.284 | -44.174 | 124.284 |
| Mn | 3.89E-02 | 1.75E-02 | 2.228 | 7.63E-02 | -5.98E-03 | 8.39E-02 | -5.98E-03 | 8.39E-02 |
| Fe* | -1.52E-03 | 6.81E-04 | -2.228 | 7.64E-02 | -3.27E-03 | 2.34E-03 | -3.27E-03 | 2.34E-03 |
| Al* | 2.93E-03 | 6.50E-03 | 4.506 | 6.37E-03 | 1.26E-03 | 1.26E-03 | 1.26E-03 | 4.60E-03 |

* DF: Degree of freedom; SQ: sum of square; MS: mean sum of square.

** SE: standard error; L: lower; U: upper

Figure S1.2: Estimated versus real concentration of Zn in samples.

Table S2: Total elemental concentration of potential toxic elements determined by X-ray fluorescence spectrometer (EDX–8000).

| **Sample** | **MgO** | **Al_2_O_3_** | **SiO_2_** | **S** | **Cl** | **CaO** | **TiO_2_** | **MnO** | **Fe_2_O_3_** | **Cu** | **Zn** | **Rb** | **Sr** | **Zr** | **Nb** | **Cd** | **Pb** |  |
| --- | --- | --- | --- | --- | --- | --- | --- | --- | --- | --- | --- | --- | --- | --- | --- | --- | --- | --- |
| **mg kg^-1^** | | | | | | | | | | | | | | | | | | |
| 1 | 12944 | 79052 | 487372 | 3872 | 1205 | 129529 | 6656 | 608 | 33237 | 73 | 238 | 109 | 410 | 297 | 24 | <LOD* | 47 |  |
| 7 | 14038 | 118459 | 510439 | 2220 | 693 | 18818 | 6257 | 2671 | 85210 | 91 | 2131 | 106 | 79 | 298 | 22 | 51 | 3198 |  |
| 8 | 9212 | 96753 | 449303 | 5583 | 3488 | 72303 | 15185 | 1373 | 58643 | 77 | 446 | 133 | 274 | 303 | 32 | <LOD | 352 |  |
| 9 | <LOD | 121263 | 466182 | 2151 | 1364 | 35289 | 6390 | 577 | 33737 | <LOD | 527 | 292 | 208 | 374 | 50 | <LOD | 106 |  |
| 11 | 8686 | 72013 | 564156 | 4065 | 1996 | 45895 | 4882 | 571 | 31351 | 57 | 72 | 79 | 170 | 193 | 24 | <LOD | 76 |  |
| 12 | 11131 | 101119 | 584616 | 1939 | 447 | 27234 | 6241 | 455 | 37222 | 19 | 68 | 85 | 101 | 329 | 19 | <LOD | 73 |  |
| 13 | 10894 | 32001 | 790545 | 1327 | <LOD | 22405 | 3226 | 278 | 9697 | 70 | <LOD | 22 | 99 | 87 | <LOD | <LOD | <LOD |  |
| 14 | 8840 | 113667 | 541465 | 3260 | 1741 | 46202 | 6817 | 544 | 29965 | 29 | 165 | 219 | 249 | 652 | 40 | <LOD | 56 |  |
| 15 | 7983 | 89587 | 532607 | 1477 | 5161 | 44625 | 7544 | 343 | 21312 | 33 | <LOD | 184 | 207 | 667 | 41 | <LOD | 51 |  |
| 16 | 10861 | 95954 | 452373 | 5907 | 3402 | 71624 | 15775 | 1235 | 59407 | 88 | 422 | 138 | 290 | 345 | 32 | <LOD | 385 |  |
| 17 | <LOD | 137550 | 510303 | 416 | <LOD | 39203 | 10520 | 634 | 56920 | 20 | 25 | 142 | 159 | 579 | 55 | <LOD | 34 |  |
| *LOD* | *7868* | *3628* | *5838* | *202* | *190* | *402* | *163* | *98* | *578* | *16* | *24* | *20* | *15* | *17* | *15* | *50* | *24* |  |
| *r^2^* | *0.991* | *0.995* | *0.995* | *-* | *-* | *0.995* | *0.998* | *0.999* | *0.999* | *1.000* | *0.998* | *0.998* | *0.999* | *0.932* | *-* | *-* | *0.999* |  |

* LOD: limit of detection

Table S3: Parameters used to assess risks to human health

| **Parameters** | **Description** | **Unit** | **Value** | **References** |
| --- | --- | --- | --- | --- |
| **IR** | Ingestion rate | mg day^-1^ | 100 for adults  200 for children | U. S. EPA, (2011) |
| **ED** | Exposure duration | Years | 24 for adults  6 for children | U. S. EPA, (2011) |
| **PE** | Exposure Frequency | Days per year | 350 | U. S. EPA, (1989) |
| **BW** | Body weight | Kg | 70 for adults  15 for children | IBGE (2010) EPA 2004 |
| **AF** | Adherence factor | mg cm^2^ h | 0.07 for adults  0.2 for children | U. S. EPA, (2002) |
| **ABS** | Dermal absorption factor | Unitless | 0.001 | ATSDR, (2016) |
| **AT** | Average exposure time | Days | ED×365 | Alsafran et al. (2021); |
| **ET** | Exposure time | h day^-1^ | 8 for adults  24 for children | U. S. EPA, (2011) |
| **PEF** | Emission factor | m^3^ kg^-1^ | 1.36x10 ^9^ | U. S. EPA, (2002) |
| **SA** | Exposed skin surface area | cm^2^ | 5700 for adults  2800 for children | Behrooz et al. (2021) |

Table S4: Concentrations of potentially toxic elements in dust samples assessed by USEPA 3051A

| Sample | **Pb** | **Zn** | **Cd** | **Cu** | **Mn** | **Ba** | **Fe** | **Al** |
| --- | --- | --- | --- | --- | --- | --- | --- | --- |
|  | **mg kg^-1^** | | | | | | **g kg^-1^** | |
| 1 | 57.75±6.2 | 240.46±14.4 | 5.96±0.9 | 55.98±2.4 | 307.8±9 | 555.36±12.1 | 34.44±0.7 | 27.92±9.3 |
| 2 | 97.4±2.4 | 243.26±3.7 | 5.93±0.8 | 61.67±1.4 | 370.25±3.2 | 489.97±23.1 | 40.24±6.2 | 36.39±5.2 |
| 3 | 108.65±0 | 402.91±0 | 6.19±0 | 30.97±0 | 327.61±0 | - | 39.18±0 | 14.59±0 |
| 4 | 103.55±6.1 | 169.57±5.4 | 5.02±1.3 | 36.67±12.3 | 297.31±40.6 | 444.11±76.4 | 99.8±3.2 | 48.52±4.2 |
| 5 | 90.74±8.9 | 319.97±71.1 | 5.33±0.9 | 23.75±4.5 | 278.03±53.4 | - | 27.3±4.1 | 20.25±5.6 |
| 6 | 112.16±30.1 | 101.61±11.5 | 5.62±0.4 | 49.02±4.5 | 259.87±39.9 | - | 51.73±11.6 | 12.81±7.1 |
| 7 | 508.04±3 | 173.63±2.6 | 36.15±0.8 | 68.64±2.9 | 824.38±38.9 | - | 47.05±8.8 | 21.05±4.0 |
| 8 | 33.53±9.3 | 60.59±3.2 | 6.75±1.6 | 22.21±1.3 | 400.33±25.6 | - | 43.52±4.1 | 15.63±1.2 |
| 9 | 38.21±0.4 | 71.18±1.4 | 6.27±0.7 | 31.64±1.2 | 518.67±15.7 | - | 63.96±1.7 | 25.79±0.3 |
| 10 | 7.37±0.6 | 48.54±1.2 | 4.26±0.9 | 33.19±0.9 | 142.84±79.6 | - | 29.36±0.8 | 8.44±0.3 |
| 11 | 47.23±0.7 | 176.72±8.1 | 6.12±1 | 34.01±3.1 | 226.01±62.7 | - | 17.21±1.8 | 10.94±1.2 |
| 12 | 21.62±1.8 | 42.36±5 | 5.65±0.4 | 7.9±0.3 | 195.74±77.7 | - | 29.12±2.4 | 18.29±1.9 |
| 13 | 22.7±0.98 | 227.4±4.8 | 6.19±0.3 | 29.21±5.1 | 283.48±16.6 | - | 8.16±0.3 | 5.92±0.3 |
| 14 | 476.03±33.5 | 365.87±13.9 | 9.96±0.1 | 60.9±2.7 | 628.36±106 | - | 76.33±3.4 | 33.43±1.7 |
| 15 | 169.86±9.7 | 198.75±12.4 | 7.05±0.9 | 67.79±1.4 | 513.33±34.1 | - | 53.84±3.4 | 22.53±1.8 |
| 16 | 170.34±8.3 | 260.61±1.2 | 12.25±3 | 61.49±3.5 | 401.48±9.2 | - | 81.34±15.9 | 15.81±2.8 |
| 17 | 836.29±22 | 866.43±16.7 | 13.86±8.2 | 74.92±0.9 | 669.04±37.3 | - | 55.04±34.9 | 22.24±7.0 |
| 18 | 90.93±14.5 | 87.01±4.5 | 6.57±0.4 | 24.62±0.4 | 266.94±14.4 | - | 22.65±3.3 | 18.82±3.3 |

Table S5: Bioaccessibility (%) of potentially toxic elements in dust samples determined with 0.43 M HNO_3_

| **HNO_3_ bioaccessibility** | | | | | | | |
| --- | --- | --- | --- | --- | --- | --- | --- |
| **Sample** | **Pb** | **Zn** | **Cd** | **Cu** | **Mn** | **Fe** | **Al** |
| 1 | 76.53 | 4.1 | 37.22 | 75.52 | 30.99 | 7.08 | 6.55 |
| 2 | 81.23 | 3.97 | 32.12 | 72.41 | 26.15 | 8.89 | 4.75 |
| 3 | 93.86 | 11.97 | 38.44 | 91.95 | 29.58 | 8.77 | 11.01 |
| 4 | 84.26 | 62.03 | 20.15 | 37.9 | 32.14 | 1.56 | 4.37 |
| 5 | 90.59 | 5.13 | 18.33 | 55.79 | 34.44 | 5.91 | 11.42 |
| 6 | 87.65 | 58.41 | 17.66 | 65.30 | 36.35 | 3.2 | 15.08 |
| 7 | 63.07 | 23.11 | 28.20 | 57.18 | 11.89 | 4.08 | 5.75 |
| 8 | 71.85 | 34.12 | 7.98 | 32.62 | 24.08 | 5.22 | 16.84 |
| 9 | 76.06 | 25.67 | 1.01 | 29.57 | 18.79 | 1.5 | 5.95 |
| 10 | 85.88 | 76.67 | 1.79 | 89.18 | 48.79 | 7.52 | 25.03 |
| 11 | 82.98 | 70.17 | 8.86 | 62.26 | 41.2 | 17.76 | 24.31 |
| 12 | 90.57 | 36.29 | 5.95 | 37.27 | 38.93 | 5.35 | 10.15 |
| 13 | 89.30 | 4.32 | 5.54 | 61.83 | 33.86 | 16.97 | 33.43 |
| 14 | 6.70 | 3.31 | 21.55 | 43.70 | 15.58 | 4.7 | 12.44 |
| 15 | 70.34 | 23.39 | 16.69 | 48.13 | 18.81 | 4.88 | 12.46 |
| 16 | 71.40 | 3.87 | 65.14 | 43.76 | 16.07 | 2.77 | 14.58 |
| 17 | 10.35 | 2.62 | 13.45 | 44.34 | 14.60 | 2.46 | 5.86 |
| 18 | 95.27 | 36.42 | 8.45 | 38.21 | 34.32 | 4.02 | 6.54 |

Table S6: Elemental ratios of dust samples and soil located near the mining tailings.

| **Samples** | **Al_2_O_3_/TiO_2_** | **SiO_2_/TiO_2_** | **TiO_2_/Zr** | **Zr/TiO_2_** | **Rb/Sr** |
| --- | --- | --- | --- | --- | --- |
| 12 | 16,20 | 93,67 | 18,95 | 0,05 | 0,84 |
| 16 | 6,08 | 28,68 | 45,74 | 0,02 | 0,48 |
| 15 | 11,87 | 70,60 | 11,31 | 0,09 | 0,89 |
| 9 | 18,98 | 72,96 | 17,08 | 0,06 | 1,40 |
| 1 | 11,88 | 73,22 | 22,40 | 0,04 | 0,27 |
| 8 | 6,37 | 29,59 | 50,06 | 0,02 | 0,49 |
| 14 | 16,67 | 79,42 | 10,45 | 0,10 | 0,88 |
| 17 | 13,08 | 48,51 | 18,18 | 0,06 | 0,90 |
| 7 | 18,93 | 81,58 | 20,99 | 0,05 | 1,34 |
| 13 | 9,92 | 245,08 | 36,96 | 0,03 | 0,22 |
| 11 | 14,75 | 115,56 | 25,26 | 0,04 | 0,46 |
|  | Average (Dust) | | | | |
|  | 13,16 | 85,35 | 25,22 | 0,05 | 0,74 |
|  | Tailing (Horizon A from a Technosol adjacent to the tailing pile) | | | | |
|  | 19,85 | 95,49 | 66,98 | 0,02 | 3,84 |

Figure S2: Cluster analysis from the collected data.

Figure S3: Data from the nearest meteorological station in Boquira, Bahia, Brazil. Meteorological station of Guanambi (A426). Source: National Meterological Institute

**References**

Serviço Geológico do Brasil (CPRM) (2014). Folha SD.23-X-B-V Boquira. Escala 1:1.000.000.

Behrooz, R. D., Kaskaoutis, D. G., Grivas, G., & Mihalopoulos, N. (2021). Human health risk assessment for toxic elements in the extreme ambient dust conditions observed in Sistan, Iran. *Chemosphere*, *262*. https://doi.org/10.1016/j.chemosphere.2020.127835
